# Dijet production in diffractive deep-inelastic scattering in next-to-next-to-leading order QCD

**DOI:** 10.1140/epjc/s10052-018-5981-z

**Published:** 2018-06-30

**Authors:** D. Britzger, J. Currie, T. Gehrmann, A. Huss, J. Niehues, R. Žlebčík

**Affiliations:** 1 Physikalisches Institut, Universität Heidelberg, Im Neuenheimer Feld 226, 69120 Heidelberg, Germany; 2 Institute for Particle Physics Phenomenology, Durham University, Durham DH1 3LE, UK; 3 Physik-Institut, Universität Zürich, Winterthurerstraße 190, 8057 Zurich, Switzerland; 4 Theoretical Physics Department, CERN, 1211 Geneva, Switzerland; 5 DESY, Notkestraße 85, 22607 Hamburg, Germany

## Abstract

Hard processes in diffractive deep-inelastic scattering can be described by a factorisation into parton-level subprocesses and diffractive parton distributions. In this framework, cross sections for inclusive dijet production in diffractive deep-inelastic electron–proton scattering (DIS) are computed to next-to-next-to-leading order (NNLO) QCD accuracy and compared to a comprehensive selection of data. Predictions for the total cross sections, 40 single-differential and four double-differential distributions for six measurements at HERA by the H1 and ZEUS collaborations are calculated. In the studied kinematical range, the NNLO corrections are found to be sizeable and positive. The NNLO predictions typically exceed the data, while the kinematical shape of the data is described better at NNLO than at next-to-leading order (NLO). A significant reduction of the scale uncertainty is achieved in comparison to NLO predictions. Our results use the currently available NLO diffractive parton distributions, and the discrepancy in normalisation highlights the need for a consistent determination of these distributions at NNLO accuracy.

## Introduction

Diffractive processes in deep-inelastic scattering, $ep \rightarrow eXY$, where the final state systems *X* and *Y* are separated in rapidity, have been studied extensively at the electron–proton collider HERA [1]. The forward system *Y* consists of the leading proton, which stays intact after the collisions, but may also contain its low mass dissociation. Between the systems *X* and *Y* a depleted region without any hadronic activity is observed, the so-called large rapidity gap (LRG). This is a consequence of the vacuum quantum numbers of the diffractive exchange which is often referred to as a pomeron ($I\!\!P$). Experimentally, the diffractive events can be selected either by requiring a rapidity region in the direction of the proton beam without any hadronic activity (LRG method) or by direct detection of the leading proton using dedicated spectrometers. In the second case, the system *Y* is free of any diffractive dissociation.

Predictions for diffractive processes in DIS can be obtained in the framework of perturbative QCD (pQCD). According to the factorisation theorem for diffractive DIS (DDIS) [2], if the process is sufficiently hard, the calculation can be subdivided into two components: the hard partonic cross sections, $\mathrm {d}\hat{\sigma }_n$, are calculable within pQCD in powers of $\alpha _{\text {s}} (\mu _{\text {R}})$, which need to be convoluted with soft diffractive parton distribution functions (DPDFs, $f^D_a$) that specify the contributing parton *a* inside the incoming hadron. DPDFs are universal for all diffractive deep-inelastic processes [2], with the hardness of the process being ensured by the virtuality $Q^2$ of the exchanged photon.

Up to now, predictions for diffractive processes, and in particular for diffractive dijet production, were performed only in next-to-leading order QCD (NLO). These predictions were able to describe the measured cross sections satisfactorily, both in shape and normalisation (for a review see e.g. Ref. [1]). However, due to their large theoretical uncertainties they did not achieve the precision of the data and thus did not allow for more stringent conclusions, i.e. about the underlying fundamental concepts of the diffractive exchange. Furthermore, the NLO predictions for dijet production were about two times higher than the leading-order (LO) predictions. This raised the natural question concerning the size of contributions from even higher orders for such processes at the comparably low scales of the HERA data.

Here, we present the next-to-next-to-leading (NNLO) perturbative QCD calculations for dijet production in diffractive DIS. These calculations are performed for the first time and constitute the first NNLO predictions for a diffractive process. We compare our predictions with several single-, double-differential and total cross sections from six distinct measurements published by the H1 or ZEUS collaboration. A quantitative comparison of NLO and NNLO predictions with the data is presented. We further study the scale dependence of the NNLO predictions. Different DPDF parameterisations are studied and we provide additional studies about the sensitivity of the dijet data for future DPDF determinations.

## NNLO predictions for dijet production in DDIS

Relevant kinematical variables to describe fully inclusive neutral current (NC) DIS can be inferred from the momenta of the incoming particles and the outgoing lepton:

$$\begin{aligned} l(k)+p(P) \rightarrow l'(k') + X(p_X), \end{aligned}$$

such that the momentum transferred to the proton is given by the momentum $q=k-k'$ of the virtual gauge boson $\gamma ^*$. The kinematics of each event is then completely determined by the following variables

1 $$\begin{aligned} s = (k+p)^2\,,\qquad Q^2 = -q^2\,,\qquad y = \frac{q\cdot p}{k\cdot p}\,, \end{aligned}$$

where *y* is referred to as the inelasticity of the scattering. Neglecting the proton mass, the $$\gamma ^*p$$ invariant squared mass is given by $$W^2=sy-Q^2 $$, and is thus directly proportional to *y* in the case $$Q^2 \ll sy$$. The variable *s* represents centre-of-mass energy squared of the *ep* collisions.

**Fig. 1:**
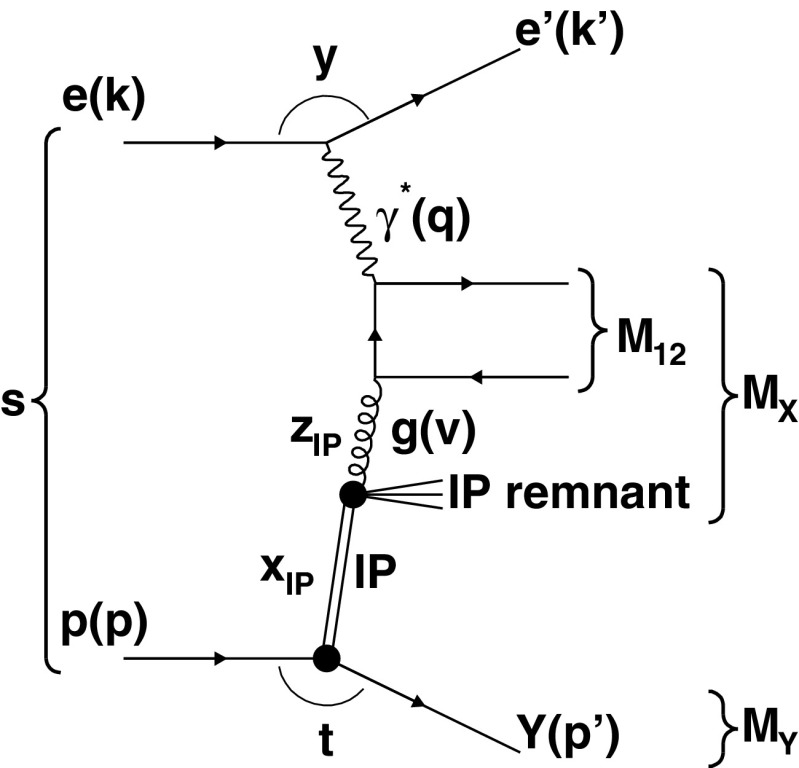
The leading order Feynman diagram for dijet production in diffractive DIS via boson-gluon fusion (taken from Ref. [3]). The variables are described in the text

The leading order Feynman diagram for dijet production in diffractive DIS is displayed in Fig. 1.

In this case, a dijet system is characterised by at least two outgoing jets within a given pseudorapidity range ($\eta _\mathrm{jet}^{*}$ or $\eta _\mathrm{lab}^\mathrm{jet}$) with sufficiently high transverse momenta $p_{\mathrm {T}}^{*,\mathrm jet}$ in the $\gamma ^*p$ rest frame.^1^ At HERA, particles are commonly clustered into jets using the $k_t$ cluster algorithm [4]. The jet with the highest (second highest) $p_{\mathrm {T}}^{*,\mathrm jet}$ is denoted as ‘leading jet’ (‘subleading jet’) and their average transverse momentum and invariant mass is calculated as $\langle p_{\mathrm {T}}\rangle =(p_{\mathrm {T}}^{*,\mathrm jet1} +p_{\mathrm {T}}^{*,\mathrm jet2})/2$ and denoted by $M_\mathrm {12}$, respectively.

For the description of the diffractive kinematics additional invariants have to be introduced and are in terms of the momentum assignments from Fig. 1 given by

2 $$\begin{aligned}&z^\mathrm{obs}_{I\!\!P} = \frac{M_\mathrm {12} ^2 + Q^2}{M_\mathrm{X}^{2} + Q^2}\,, \quad x_{I\!\!P} = \frac{(p - p')\cdot q}{p\cdot q},\nonumber \\&\quad \quad \mathrm{and}\quad t = (p-p')^2\,. \end{aligned}$$

The observable $z^\mathrm{obs}_{I\!\!P}$ is calculated from $M_\mathrm {12}$ and the invariant mass of the hadronic system *X*, $M_\mathrm{X}$, and it characterises the parton momentum fraction of the diffractive exchange entering the partonic sub-process.^2^ The denominator in the definition of $z^\mathrm{obs}_{I\!\!P}$ can equivalently be written as $x_{I\!\!P} ys$, i.e. in terms of kinematic variables related to the scattered electron and the leading proton. The observable $x_{I\!\!P}$ is interpreted as the relative energy loss of the leading proton and is given by $x_{I\!\!P} = 1-{E_{p'}}/{E_p}$. For measurements at HERA, $x_{I\!\!P}$ is typically of $\mathcal {O}(0.01)$. The variable *t* is related to the transverse momentum of the diffractive proton ($t \simeq -p^2_{\mathrm{T},p'}$) with absolute value $\sim \! 0.1\,\text {GeV}^2$ at HERA. The mass of the system *Y*, which is formed by either a leading proton or its low mass dissociative state, is denoted as $M_\mathrm{Y}$.

QCD predictions for sufficiently hard processes in diffractive DIS are obtained by subdividing the calculation into two parts in accordance with the factorisation theorem [2]: The calculation of the hard partonic scattering coefficients, $\mathrm {d}\hat{\sigma }_i$, that are calculable within pQCD and come with the $i^{th}$ power of $\alpha _{\text {s}} (\mu _{\text {R}})$, and the convolution of the $\mathrm {d}\hat{\sigma }_i$ with appropriate DPDFs that capture the properties of the soft physics, denoted by $f^D_a$ for incoming parton of type *a*. The full cross section up to power *n* in $\alpha _{\text {s}} (\mu _{\text {R}})$ can then be written as a sum over the relevant hard coefficients and partonic channels,

3 $$\begin{aligned} \sigma _n = \sum _{a=g,q,\bar{q}}\sum _{i=1}^n\sigma _{a,i}\,. \end{aligned}$$

In the above, the function $\sigma _{a,i}$ is calculated as a convolution of the DPDFs with the hard coefficients:

4 $$\begin{aligned} \sigma _{a,i}= & {} \int \mathrm {d}t \int \mathrm {d}x_{I\!\!P} \int \mathrm {d}z_{I\!\!P} \,\,\mathrm {d}\hat{\sigma }_i^{ea\rightarrow 2 \mathrm{jets}} (\hat{s},\mu _{\text {R}},\mu _{\text {F}})\nonumber \\&\times f^{D}_{a}(z_{I\!\!P},\mu _{\text {F}}, x_{I\!\!P},t). \end{aligned}$$

Physically, the variable $x_{I\!\!P}$ represents the longitudinal proton momentum fraction which contributes to the interaction or, alternatively, the momentum fraction the proton is loosing in the diffractive exchange. The variable $z_{I\!\!P}$ is then the fraction of the diffractive exchange momentum which enters the hard subprocess, where it should be noted that the variable $z_{I\!\!P}$ equals $z^\mathrm{obs}_{I\!\!P}$ only at leading order. The variable $\hat{s}$ denotes the centre-of-mass energy squared of the underlying parton–electron interaction and can be expressed as $\hat{s} = x_{I\!\!P} z_{I\!\!P} s$.

The DPDFs have many properties similar to the non-diffractive PDFs, in particular they obey the DGLAP evolution equation [2, 5–7], however, DPDFs are constrained by the presence of the leading proton in the final state. In parameterised DPDFs the *t*-dependence of the cross section is integrated out and in the considered measurements is restricted either by $|t|<1\,\mathrm GeV ^2$ or $|t|<0.6\,\mathrm GeV ^2$.

In this paper, the parton-level jet-production cross sections in DDIS are calculated up to NNLO. These calculations are identical to the NNLO calculations in the non-diffractive case [8, 9]. The NNLO correction involves three types of scattering amplitudes: the two-loop amplitudes for two-parton final states [10–13], the one-loop amplitudes for three-parton final states [14–17] and the tree-level amplitudes for four-parton final states [18–20]. These contributions contain implicit infrared divergences from soft and/or collinear real-emission corrections as well as explicit divergences of both infrared and ultraviolet origin from the virtual loop corrections. When calculating predictions for an infrared-safe final state definition, these singularities cancel when the different parton multiplicities are combined [21]. The calculation employs the antenna subtraction method [22–25]: For real-radiation processes, the subtraction terms are constructed out of antenna functions, which encapsulate all color-ordered unresolved parton emission in-between pairs of hard radiator partons. To constitute a subtraction term, the antenna functions are then multiplied with reduced matrix elements of lower partonic multiplicity. By making the infrared pole structure explicit, the integrated subtraction terms can be combined with the virtual corrections in order to obtain a finite result. Relevant tree-level and one-loop matrix elements were verified against Sherpa [26–28] and nlojet++ [29–31]. Our computation is performed within the parton-level event generator NNLOJET [32], which implements the antenna subtraction formalism and further provides a validation framework to ensure the correctness of the results. These tests comprise the analytic cancellation of all infrared poles and a numerical check of the behaviour of the subtraction terms to mimic the real-emission matrix elements in all unresolved limits [33, 34]. All calculations are performed using the $\overline{\mathrm{MS}}$ renormalisation scheme and for five massless quark flavors. The strong coupling constant is set to $\alpha _{\text {s}} (M_{\text {Z}}) =0.118$ [35].

The calculation of NNLO partonic cross sections [8, 9] has recently been applied successfully to describe inclusive jet and dijet cross section data in non-diffractive DIS [8, 9, 36, 37]. Here, however, the hard coefficients are now convoluted with DPDFs for the first time and we present the first calculation of a diffractive jet production process to NNLO in $\alpha _{\text {s}} (\mu _{\text {R}})$. For this reason our predictions are limited by the available DPDFs which have only been determined up to NLO so far.

For the convolutions with the DPDFs, the phase space integration of the matrix element squared has to be adopted for the integrations over the additional diffractive variables *t* and $x_{I\!\!P}$. This has been reported, for instance, for implementations in the programs DISENT [38, 39], JetViP [40–42] and nlojet++ [29, 30, 43]. While these previous calculations commonly used the computationally very expensive Monte Carlo or the slicing method [43], here an improved convolution formalism is used. Our calculation thereby employs the fastNLO formalism [3, 44, 45] which has the advantage that the matrix elements have to be calculated only once and can then be used repeatedly for integrations of the DPDFs. The formalism will be briefly explained in the following.

The matrix elements $\mathrm {d}\hat{\sigma }_n^{ea\rightarrow 2 \mathrm{jets}}$ have their $x_{I\!\!P}$ and $z_{I\!\!P}$ dependence given through $\hat{s}= xs$, where *s* is the centre-of-mass energy squared of the *ep* collision and the momentum fraction *x* is given by

5 $$\begin{aligned} x = x_{I\!\!P} z_{I\!\!P} \,. \end{aligned}$$

In the fastNLO approach for non-diffractive DIS the *x*-dependence of the matrix elements is frozen on a grid,

6 $$\begin{aligned} \int \mathrm {d}x\, \mathrm {d}\hat{\sigma }_{a,n}(x)\, f(x) \simeq \sum _i \tilde{\sigma }_i^{(a,n)} f(x_i), \end{aligned}$$

where the nodes lie at set values of $x_i$. With an increasing number of nodes the approximation improves until both expressions in Eq. (6) become numerically identical. The coefficients $\tilde{\sigma }_i$ are calculated from contributing matrix elements for a given measurement function, which expresses the given observable, phase space and jet definition. While this calculation is computationally very expensive, it has to be performed only once, since these coefficients are independent of PDF values (DPDFs) and scales.

Using Eq. (6) the partonic cross section in DDIS Eq. (4) is then calculated as

7 $$\begin{aligned} \sigma _{a,n} = \int \mathrm {d}t\int \tfrac{\mathrm {d}x_{I\!\!P}}{x_{I\!\!P}} \sum _i^{x_i<x_{I\!\!P}} \tilde{\sigma }_i^{(a,n)} {f}^D_a(x_{I\!\!P},z_{I\!\!P} =x_i/x_{I\!\!P},t)\,. \end{aligned}$$

By interpreting the factor $1/x_{I\!\!P}$ as the flux factor of the diffractive exchange, then according to a center-of-mass reweighting of the incoming hadron, the calculation can be made equivalent to the slicing method. Our calculations have been validated in NLO accuracy against calculations using nlojet++ [30, 31] with the slicing method.

The fastNLO based approach has advantages of a higher numerical accuracy of the $x_{I\!\!P}$ integration, and, more importantly still, a significantly higher numerical accuracy is achieved in the calculation of the hard matrix elements for a given amount of computing time. This is of great importance for the calculation of the double-real and real-virtual NNLO amplitudes, which are calculated here using several 100,000 hours of CPU time using state-of-the-art CPUs. The numerical accuracy of the fastNLO interpolation technique is typically smaller than the numerical precision of the tabulated DPDFs, and thus can be neglected.

In order to avoid regions of the phase space where the predictions exhibit an enhanced infrared sensitivity [41, 46], the phase space definitions of all analyses have asymmetric cuts on the transverse momenta of the two leading jets. It was tested that the difference of $\sim \! 1\, \text {GeV}$ between the cuts on the leading and sub-leading jet is sufficient to remove this region.

For the nominal calculations the renormalisation ($\mu _{\text {R}}$) and factorisation scale ($\mu _{\text {F}}$) are set to

8 $$\begin{aligned} \mu _{\text {R}} ^2=\mu _{\text {F}} ^2=Q^2 +\langle p_{\mathrm {T}}\rangle ^2\,, \end{aligned}$$

while also different choices are studied. The ‘scale’ uncertainty of the prediction is obtained by varying $\mu _{\text {R}}$ and $\mu _{\text {F}}$ by the conventional factors of 0.5 and 2.

Diffractive parton distributions are determined 3 by interpreting data for different final states in DDIS in a parton model framework [48]. Already the first inclusive DDIS data from HERA [49] indicated the presence of a very large gluon content in the diffractive exchange [50]. The knowledge of the DPDFs is at a lower precision than that of non-diffractive PDFs. This is due to the uncertainties of the DDIS measurements, but also because available data sets are not always compatible [51]. In addition, different assumptions imposed for their determination result in substantial differences of individual DPDFs. Therefore, different DPDF sets may result in sizeable differences for certain processes and kinematic regions. Currently, all DPDFs available have been obtained using data together with corresponding NLO QCD predictions only. Given the typical scales of the HERA measurements, higher order QCD effects are sizable and NNLO DPDFs are expected to differ significantly from their NLO variants. Nonetheless, due to the absence of NNLO DPDFs we have to use NLO DPDFs and the following sets are studied:

- H1FitB  [52] is the most widely used DPDF. It was determined from an NLO DGLAP QCD fit to reduced inclusive DDIS cross sections. The diffractive data was selected using the LRG method and, therefore, the DPDF includes proton dissociation into a low-mass hadronic state ($M_\mathrm{Y} <1.6\,\mathrm GeV$). The phase space of the selected data was restricted to $\beta < 0.8$ and $Q^2 >8.5\,\mathrm GeV ^2$. The gluon DPDF at the starting scale of the evolution, $\mu _0^2 = 1.75\,\mathrm GeV ^2$, was assumed to be a constant, i.e. independent of the value of $z_{I\!\!P}$.
- H1FitA  [52] is a variant of the H1FitB DPDF, which uses a more flexible parameterisation of the gluon distribution at the starting scale of the evolution. In comparison to the H1FitB DPDF, a significantly larger gluon DPDF is found although both, the H1FitA and the H1FitB DPDF, describe the shape of the data equally well, as inclusive DDIS cross sections are only weakly sensitive to the gluon DPDF. A detailed analysis of dijet data suggests [43] that the gluon component in the H1FitA DPDF is overestimated.
- H1FitJets  [43] is the first DPDF fitted based on the combination of inclusive and dijet data, using the same inclusive data sample as for H1FitB and H1FitA. The inclusion of dijet data, which is more sensitive to the gluon content, led to a slightly smaller gluon distribution compared to the H1FitB DPDF.
- ZEUSSJ  [53] is determined by a combined fit of inclusive and dijet data by the ZEUS collaboration. Compared to H1 fits, the proton dissociation has been subtracted using Monte Carlo (MC) estimates such that this DPDF is defined for elastic scattering ($M_\mathrm{Y} =m_P$).
- The MRW DPDF [54] is based on the same data as the H1FitB DPDF. In contrast, however, Regge factorisation is only assumed at the starting scale and the evolution is performed using inhomogeneous evolution equations accounting for pomeron-to-parton splittings.

The DPDF uncertainty in our calculations is obtained from the error sets provided together with the H1FitB DPDF. The very recent GKG18 DPDF [55], which is also in NLO, is not considered in this analysis.

Similarly as in the definitions for DPDF fits, also the various measurements impose different definitions of $M_\mathrm{Y}$. The LRG measurements by H1 are defined for $M_\mathrm{Y} <1.6\,\mathrm GeV$, whereas ZEUS extrapolated its LRG measurement to $M_\mathrm{Y} =m_P$. Two of the H1 measurements are based on proton spectrometers (FPS, VFPS), and thus these data do not contain any proton dissociation ($M_\mathrm{Y} =m_P$).

In order to provide predictions for all of the measured cross section data with any of the available DPDF sets, correction factors for proton dissociation have to be applied wherever applicable. The latest value of the proton dissociation fraction for the phase space imposed by H1 was estimated to be [56]

9 $$\begin{aligned} \frac{\sigma ( M_\mathrm{Y} < 1.6\,\text {GeV})}{\sigma ( M_\mathrm{Y} = m_P)} = 1.20 \pm 0.11 (\mathrm {exp.})\,. \end{aligned}$$

This value was obtained as a combination of the previously measured value of $1.23 \pm 0.16$ [57] and a newly measured value of $1.18 \pm 0.12$ . It is consistent with the prediction of 1.15  obtained with the DIFFVM generator [58].

In order to compare the data with fixed-order predictions, correction factors accounting for hadronisation effects are applied. These are estimated using MC simulations and corresponding correction factors are provided together with the respective data as discussed in the next section.

## Data sets and observables

The NNLO cross sections are computed for six measurements taken at HERA by the H1 or ZEUS collaborations. We will refer to them as

- H1 FPS (HERA 2)  [59],
- H1 VFPS (HERA 2)  [60],
- H1 LRG (HERA 2)  [3],
- H1 LRG (HERA 1)  [43],
- H1 LRG ($$300\,\mathrm GeV $$)  [61], and
- ZEUS LRG (HERA 1)  [62].

Five of those are performed at a centre-of-mass energy of $\sqrt{s}=319\,\mathrm GeV$, and one at $\sqrt{s}=300\,\mathrm GeV$ [61], depending on the proton beam energy of 920 or 820 $\mathrm GeV$, respectively, while the electron or positron beam energy was always equal to 27.6 $\mathrm GeV$. In two cases the leading proton is identified by the forward proton spectrometer (FPS) [59] or very forward proton spectrometer (VFPS) [60], otherwise the diffractive events are selected using the LRG method. Jets were identified using the $k_T$ jet algorithm in the $\gamma ^*p$ frame with cone parameter $R=1$, and at least two jets are required in each event. The phase space definitions of the measurements are summarised in Table 1. The hadronisation corrections are provided together with the data [3, 43, 59–61], or in case of Ref. [62], are displayed in Ref. [63]. Dijet cross sections are studied differentially in several kinematic variables, which also constrain the phase space of the measurements, and their meanings are described in Fig. 1.

**Table 1:** Summary of the dijet data sets. The first column represents the data set label and the second shows the integrated luminosity and the number of events of the given data set. The other columns summarise the definition of the phase space of the given data. In cases, where the DIS phase space is defined in terms of *W*, the corresponding range in $y=W^2/s$ is shown. All measurements have in common a requirement of $n_\mathrm{jets} \ge 2$ in the given dijet range, which is applied after identifying the two leading jets

Data set	$\varvec{\mathcal {L}}$	DIS range	Dijet range	Diffractive range
H1 FPS (HERA 2) [59]	156.6 $\mathrm{pb}^{-1}$	$4<Q^2 <110\,\mathrm GeV ^2$	$p_{\mathrm {T}}^{*,\mathrm jet1} >5\,\mathrm GeV$	$x_{I\!\!P} <0.1$
	(581ev)	$0.05< y < 0.7$	$p_{\mathrm {T}}^{*,\mathrm jet2} >4.0\,\mathrm GeV$	$|t|<1\,\mathrm GeV ^2$
			$-1<\eta _\mathrm{lab}^\mathrm{jet} <2.5$	$M_\mathrm{Y} = m_P$
H1 VFPS (HERA 2) [60]	50 $\mathrm{pb}^{-1}$	$4<Q^2 <80\,\mathrm GeV ^2$	$p_{\mathrm {T}}^{*,\mathrm jet1} >5.5\,\mathrm GeV$	$0.010<x_{I\!\!P} <0.024$
	(550ev)	$0.2< y < 0.7$	$p_{\mathrm {T}}^{*,\mathrm jet2} >4.0\,\mathrm GeV$	$|t|<0.6\,\mathrm GeV ^2$
			$-1<\eta _\mathrm{lab}^\mathrm{jet} <2.5$	$M_\mathrm{Y} = m_P$
H1 LRG (HERA 2) [3]	290 $\mathrm{pb}^{-1}$	$4<Q^2 <100\,\mathrm GeV ^2$	$p_{\mathrm {T}}^{*,\mathrm jet1} >5.5\,\mathrm GeV$	$x_{I\!\!P} <0.03$
	($\sim$15000ev)	$0.1< y < 0.7$	$p_{\mathrm {T}}^{*,\mathrm jet2} >4.0\,\mathrm GeV$	$|t|<1\,\mathrm GeV ^2$
			$-1<\eta _\mathrm{lab}^\mathrm{jet} <2$	$M_\mathrm{Y} <1.6 \, \mathrm GeV$
H1 LRG (HERA 1) [43]	51.5 $\mathrm{pb}^{-1}$	$4<Q^2 <80\,\mathrm GeV ^2$	$p_{\mathrm {T}}^{*,\mathrm jet1} >5.5\,\mathrm GeV$	$x_{I\!\!P} <0.03$
	(2723ev)	$0.1< y < 0.7$	$p_{\mathrm {T}}^{*,\mathrm jet2} >4.0\,\mathrm GeV$	$|t|<1\,\mathrm GeV ^2$
			$-3<\eta ^{*\mathrm {jet}}<0$	$M_\mathrm{Y} <1.6 \, \mathrm GeV$
H1 LRG ($300\,\mathrm GeV$) [61]	18 $\mathrm{pb}^{-1}$	$4<Q^2 <80\,\mathrm GeV ^2$	$p_{\mathrm {T}}^{*,\mathrm jet1} >5\,\mathrm GeV$	$x_{I\!\!P} <0.03$
	(322ev)	$165< W < 242\,\mathrm GeV$	$p_{\mathrm {T}}^{*,\mathrm jet2} >4.0\,\mathrm GeV$	$|t|<1\,\mathrm GeV ^2$
		$(0.30< y < 0.65)$	$-1<\eta _\mathrm{lab}^\mathrm{jet} <2$	$M_\mathrm{Y} <1.6 \, \mathrm GeV$
			$$-3<\eta ^{*\mathrm {jet}}<0$$	
ZEUS LRG (HERA 1) [62]	61 $\mathrm{pb}^{-1}$	$5<Q^2 <100\,\mathrm GeV ^2$	$p_{\mathrm {T}}^{*,\mathrm jet1} >5\,\mathrm GeV$	$x_{I\!\!P} <0.03$
	(5539ev)	$100< W < 250\,\mathrm GeV$	$p_{\mathrm {T}}^{*,\mathrm jet2} >4.0\,\mathrm GeV$	$|t|<1\,\mathrm GeV ^2$
		$(0.10< y < 0.62)$	$-3.5<\eta ^{*\mathrm {jet}}<0$	$M_\mathrm{Y} = m_P$

Measurements were performed as functions of:

- The DIS kinematic variables: $Q^2$, *y* and *W*;
- The jet transverse momentum observables: $p_{\mathrm {T}}^{*,\mathrm jet1}$, $p_{\mathrm {T}}^{*,\mathrm jet2}$, $\langle p_{\mathrm {T}}\rangle$ and $p_{\mathrm {T}}^{*,\mathrm jet}$. Here $p_{\mathrm {T}}^{*,\mathrm jet}$ refers to the $p_\mathrm {T}$ of the leading and subleading jet;
- The jet pseudorapidity observables: $\langle \eta _\mathrm{lab}^\mathrm{jet}\rangle$, $\eta _\mathrm{jet}^{*}$, $\Delta \eta _\mathrm{lab}^\mathrm{jet}$, and $\Delta \eta ^{*}$. Here $\langle \eta _\mathrm{lab}^\mathrm{jet}\rangle$ denotes the average pseudorapidity $\eta _\mathrm{jet}^{*}$ of the two leading jets and $\Delta \eta _\mathrm{lab}^\mathrm{jet}$ and $\Delta \eta ^{*}$ denote their separation in pseudorapidity;
- Observables of the diffractive final state: $x_{I\!\!P}$, $z^\mathrm{obs}_{I\!\!P}$ and $M_\mathrm{X}$;
- Double-differential measurements as functions of $z^\mathrm{obs}_{I\!\!P}$ or $p_{\mathrm {T}}^{*,\mathrm jet1}$ for $Q^2$ intervals, and as a function of $z^\mathrm{obs}_{I\!\!P}$ for $p_{\mathrm {T}}^{*,\mathrm jet1}$ intervals.

In the fastNLO approach, the $\tilde{\sigma }_i^{(a,n)}$ coefficients are calculated prior to the convolution with the DPDFs. In this first step, however, only observables that are accessible from information on the final state kinematics of the hard matrix element can be evaluated directly. An example for such variables are the DIS kinematic variables or jet momenta. In contrast, the kinematics of the hard matrix elements do not depend explicitly on the outgoing proton momentum. Observables depending on the diffractive final state have therefore to be derived in additional steps when the $x_{I\!\!P}$ and |*t*| integration is performed (c.f. Eq. (7)). In such cases (for instance for the $x_{I\!\!P}$ and |*t*| observables), differential predictions are obtained from $\tilde{\sigma }_i^{(a,n)}$ coefficients representing the total hard cross section. Similarly, predictions as a function of $z^\mathrm{obs}_{I\!\!P}$ are calculated using the relation $z^\mathrm{obs}_{I\!\!P} =\xi /x_{I\!\!P}$ and are obtained from $\tilde{\sigma }_i^{(a,n)}$ coefficients for a highly resolved distribution in $\xi$, which denotes the proton momentum fraction carried by the incoming parton at leading order and is calculated as $\xi =x_{\mathrm {Bj}} (1+M_\mathrm {12} ^2/Q^2)$ [9]. Predictions as a function of $M_\mathrm{X}$ are obtained using the $\tilde{\sigma }_i^{(a,n)}$ coefficients for a highly resolved distributions in *y* and $Q^2$, in combination with $M_\mathrm{X} = \sqrt{ysx_{I\!\!P}-Q^2}$.

## Results

### Total dijet production cross section

**Fig. 2:**
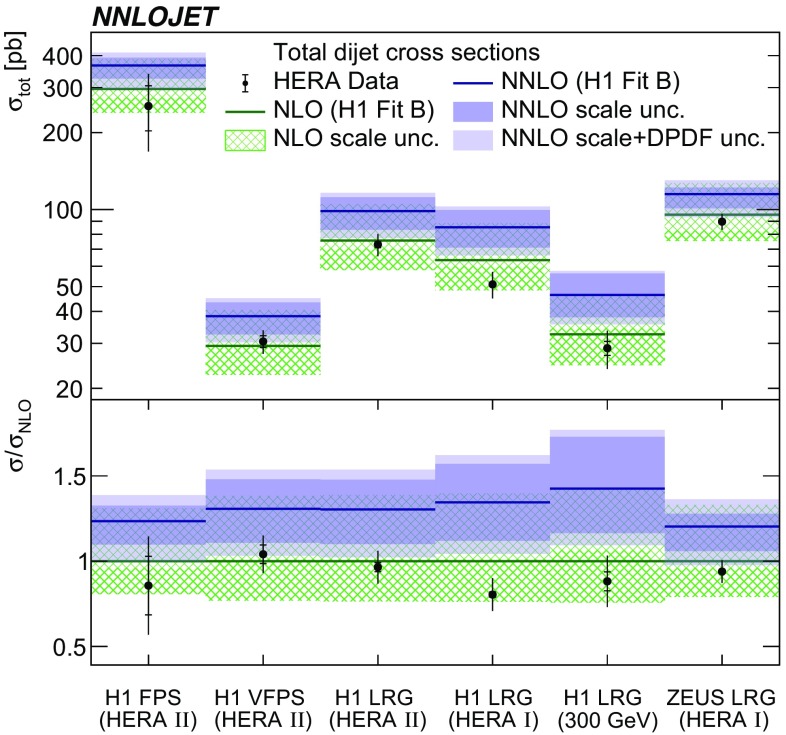
The comparison of the QCD predictions at NLO and NNLO for the total dijet cross sections with the measurements. The inner data error bars represent statistical uncertainties and other error bars are statistical and systematic errors added in quadrature. The theoretical predictions using H1FitB are displayed together with their scale uncertainties (NLO and NNLO) and with scale and DPDF uncertainties added in quadrature (only NNLO). The lower panel displays the ratio to the NLO predictions

The NNLO predictions for the total dijet cross sections of the six different experimental measurements are presented in Table 2 and are graphically displayed in Fig. 2. In both, results for the corresponding measured cross sections as well as for the NLO predictions are also included.

**Table 2:** Comparison of the measured and predicted total dijet cross sections for the six measurements. Listed are the data cross section, $\sigma ^\mathrm{Data}$, the NLO and the NNLO predictions, $\sigma ^\mathrm{NLO}$ and $\sigma ^\mathrm{NNLO}$, respectively. For $\sigma ^\mathrm{Data}$ the uncertainties denote the statistical and the systematic uncertainty. In case of H1 LRG ($300\,\mathrm GeV$), the total cross section is calculated by us from the single-differential distributions. The uncertainty of the NLO or NNLO predictions denote the scale uncertainty obtained from a simultaneous variation of $\mu _{\text {R}}$ and $\mu _{\text {F}}$ by factors of 0.5 and 2. The last two columns show the DPDF uncertainty obtained from H1FitB for the NLO or NNLO predictions. In terms of a relative uncertainty, the DPDF uncertainty is almost identical for NLO and NNLO predictions

Data set	$\sigma ^\mathrm{Data}$	$\sigma ^\mathrm{NLO}$	$\sigma ^\mathrm{NNLO}$	$\Delta _\mathrm{DPDF}^\mathrm{NLO}$	$\Delta _\mathrm{DPDF}^\mathrm{NNLO}$
	[pb]	[pb]	[pb]	[pb]	[pb]
H1 FPS (HERA 2)	$254\pm 20\pm 27$	$296^{+92}_{-57}$	$366^{+27}_{-41}$	$^{+29}_{-46}$	$^{+36}_{-57}$
H1 VFPS (HERA 2)	$30.5\pm 1.6\pm 2.8$	$29.3^{+11.2}_{-6.7}$	$38.3^{+5.1}_{-5.8}$	$^{+3.2}_{-4.2}$	$^{+4.4}_{-5.6}$
H1 LRG (HERA 2)	$73\pm 2\pm 7$	$75.7^{+29.4}_{-17.7}$	$98.6^{+13.2}_{-15.4}$	$^{+8.5}_{-10.9}$	$^{+11.7}_{-14.7}$
H1 LRG (HERA 1)	$51\pm 1^{+7}_{-5}$	$63.4^{+25.2}_{-15.1}$	$85.3^{+14.3}_{-14.3}$	$^{+7.1}_{-9.2}$	$^{+10.1}_{-12.7}$
H1 LRG (300 Gev)	$28.7\pm 1.8 \pm 3.0$	$32.5^{+13.7}_{-7.9}$	$46.4^{+9.9}_{-8.5}$	$^{+3.5}_{-4.6}$	$^{+5.3}_{-6.7}$
ZEUS LRG (HERA 1)	$89.7\pm 1.2^{+6.0}_{-6.4}$	$95.5^{+31.5}_{-20.0}$	$114.9^{+7.1}_{-13.8}$	$^{+10.5}_{-13.4}$	${}^{+13.5}_{-16.7}$

The NNLO predictions compared to the NLO predictions are higher by about 20–40 %. Since the kinematic ranges of different measurements are rather similar (Table 1), also the NNLO corrections are of similar size for the individual measurements. As found previously [3, 43, 59–61], the NLO predictions provide a good description for all of the data. In contrast, the NNLO predictions typically overshoot the data. This tension between NNLO and data may be attributed to inappropriate DPDFs, where we use the H1FitB DPDF set, which has been determined using NLO predictions. In particular, the gluon component in this DPDF appears to be too high for the usage with NNLO QCD coefficients, as this DPDF has been determined from inclusive DDIS cross section data using the respective NLO predictions only.

When compared to our common predictions, all measurements appear to be consistent with each other, although they use different techniques for the identification of the diffractive final states.

### NNLO scale uncertainty and scale choice

The scale uncertainties, which are obtained by a simultaneous variation of $\mu _{\text {R}}$ and $\mu _{\text {F}}$ by factors of 0.5 and 2, are found to be reduced significantly for NNLO predictions in comparison to NLO predictions (see also Table 2 and Fig. 2). The typical size of the scale uncertainty of the total dijet cross sections at NNLO is about 15 %, whereas it is about 35 % in NLO. In case of the H1 LRG (HERA 2) total cross section for instance, the upward (downward) scale uncertainty is reduced from 39 % (23 %) at NLO to 13 % (16 %) at NNLO. This makes these uncertainties competitive with the data uncertainty ($\sim \! 10\%$). For all total cross section measurements, however, the differences between data and NNLO predictions are larger than respective theoretical scale uncertainties.

**Fig. 3:**
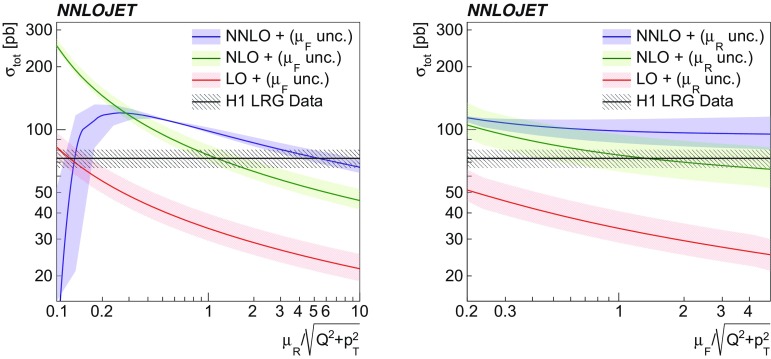
The dependence of the total dijet cross section of the H1 LRG (HERA 2) analysis on the renormalisation (left) and factorisation (right) scale. The left (right) panel displays a variation of $\mu _{\text {R}}$ ($\mu _{\text {F}}$) by factors between 0.1 and 10 and the effect of the variation of $\mu _{\text {F}}$ ($\mu _{\text {R}}$) with factors of 0.5 and 2 is displayed by the shaded areas. The calculated cross sections are shown at LO, NLO and NNLO accuracy. The measured data cross section with its total uncertainty is displayed as a black line and hatched area

A detailed investigation of the scale dependence of the LO, NLO and NNLO predictions is displayed in Fig. 3 for the H1 LRG (HERA 2) phase space. While the NLO scale dependence is of similar size as for LO predictions, the scale dependence of the NNLO predictions is significantly reduced. The $\mu _{\text {R}}$ dependence is significantly larger than the $\mu _{\text {F}}$ dependence, which is also found for non-diffractive jet production [37]. The K-factor of the NNLO correction (defined as $\sigma _\mathrm{NNLO}/\sigma _\mathrm{NLO}$) is found to be significantly smaller than the K-factor of the NLO corrections ($\sigma _\mathrm{NLO}/\sigma _\mathrm{LO}$), thus indicating convergence of the perturbative series. In comparison to data, the NNLO predictions exceed the H1 LRG (HERA 2) data for a wide range of scale factors.

**Fig. 4:**
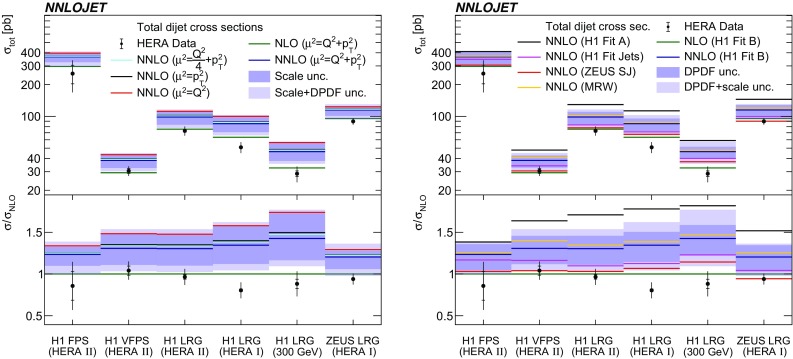
The comparison of the NNLO predictions for the total dijet cross sections with the measurements and NLO predictions. The dark shaded bands display the scale (left) and DPDF uncertainties (right), and the light shaded bands display these uncertainties added in quadrature. The left panel displays NNLO predictions for different scale definitions. The right panel displays NNLO predictions for different DPDF choices. The lower panels display the ratio to NLO predictions

The NNLO calculations are repeated for alternative choices for $$\mu _{\text {R}} ^2$$ and $$\mu _{\text {F}} ^2$$ using $$\tfrac{Q^2}{4}+\langle p_{\mathrm {T}}\rangle ^2$$, $$\langle p_{\mathrm {T}}\rangle ^2$$ and $$Q^2 $$, and results are displayed in Fig. 4 (left). Numerical values for the phase space of the H1 LRG (HERA 2) analysis are listed in Table 3. The cross sections obtained with scale choices involving $$\langle p_{\mathrm {T}}\rangle ^2$$ in their definitions differ only moderately among each other. In contrast, a scale choice of $$\mu ^2=Q^2 $$ changes the predictions significantly compared to the aforementioned scale choices. In this case, the differences are of similar size to the scale uncertainties. This can be traced back to kinematic regions where $$Q^2$$ is small compared to $$\langle p_{\mathrm {T}}\rangle ^2$$, and a choice of $$Q^2$$ can be considered as inappropriate.

**Table 3:** NNLO predictions for H1 LRG (HERA 2) using different choices for $\mu _{\text {R}} ^2$ and $\mu _{\text {F}} ^2$. The uncertainties denote the scale uncertainty from simultaneously varying $\mu _{\text {R}}$ and $\mu _{\text {F}}$ by factors of 0.5 or 2

Data set	$$\sigma ^\mathrm{Data}$$	$$Q^2 +\langle p_{\mathrm {T}}\rangle ^2$$	$$Q^2 $$	$$\langle p_{\mathrm {T}}\rangle ^2$$	$$\tfrac{Q^2}{4}+\langle p_{\mathrm {T}}\rangle ^2$$	$$\sqrt{Q^4+\langle p_{\mathrm {T}}\rangle ^4}$$
	[pb]	[pb]	[pb]	[pb]	[pb]	[pb]
H1 LRG (HERA 2)	$73\pm 7_\mathrm{exp}$	$98.6^{+13.2}_{-15.4}$	$111.7^{-43.4}_{-11.5}$	$102.1^{+8.4}_{-15.2}$	$101.1^{+10.6}_{-15.4}$	$101.0^{+11.2}_{-15.5}$

### DPDF choice and uncertainties

In Fig. 4 (right), we study the dependence of the total cross sections on the choice of DPDFs, using H1FitA  [52], H1FitB  [52], H1FitJets  [43], MRW  [54] and ZEUSSJ  [53] DPDFs. Numerical values for the H1 LRG (HERA 2) phase space are provided in Table 4. The NNLO predictions overshoot the data for any choice of DPDFs. However, it is observed that DPDFs that also consider dijet data in their determination [43, 53] (using dijet NLO predictions) give smaller predictions than DPDFs that depend on inclusive DDIS data only [52]. The differences between the predictions are mostly covered by the DPDF uncertainties of H1FitB. The DPDF H1FitA  [52] predicts a much larger cross section and thus appears to overestimate the gluon component significantly. It must be noted again that due to the absence of suitable DPDFs to NNLO accuracy, only DPDFs which have been determined to NLO accuracy could be used for our predictions. A consistent treatment of higher order contributions to the hard matrix elements for all processes entering the fits of DPDFs will enable their consistent determination to NNLO. It is considered to be of crucial importance for future improvements for predictions of DDIS processes.

**Table 4:** NNLO predictions for H1 LRG (HERA 2) using different DPDFs. Mind, all DPDFs have been determined only in NLO accuracy. The uncertainties denote the DPDF uncertainty as provided by the respective DPDF sets

Data set	$\sigma ^\mathrm{Data}$	$\sigma ^{\text{ H1FitA }}$	$\sigma ^{\text{ H1FitB }}$	$\sigma ^{\text{ H1FitJets }}$	$\sigma ^{\text{ MRW }}$	$\sigma ^{\text{ ZEUSSJ }}$
	[pb]	[pb]	[pb]	[pb]	[pb]	[pb]
H1 LRG (HERA 2)	$73\pm 7_\mathrm{exp}$	$129.3^{+16.8}_{-20.4}$	$98.6^{+11.7}_{-14.7}$	83.1	101.8	78.0

**Table 5:** Overview of the measured single- and double-differential distributions

Histogram	H1 FPS	H1 VFPS	H1 LRG	H1 LRG	H1 LRG	ZEUS LRG
	(HERA 2)	(HERA 2)	(HERA 2)	(HERA 1)	($300\,\mathrm GeV$)	(HERA 1)
$$Q^2 $$	$$\checkmark $$	$$\checkmark $$	$$\checkmark $$		$$\checkmark $$	$$\checkmark $$
$y ~[W]^*$	$\checkmark$	$\checkmark$	$\checkmark$	$\checkmark$	$*$	$*$
$p_{\mathrm {T}}^{*,\mathrm jet1} ~[p_{\mathrm {T}}^{*,\mathrm jet} ]^*$	$\checkmark$	$\checkmark$	$\checkmark$	$\checkmark$	$\checkmark$	$*$
$\langle p_{\mathrm {T}}\rangle$			$\checkmark$			
$p_{\mathrm {T}}^{*,\mathrm jet2}$			$\checkmark$			
$\langle \eta _\mathrm{lab}^\mathrm{jet}\rangle ~[\eta _\mathrm{jet}^{*} ]^*$		$\checkmark$			$\checkmark$	$*$
$\Delta \eta _\mathrm{lab}^\mathrm{jet} ~[\Delta \eta ^{*} ]^*$	$*$	$\checkmark$	$*$	$*$	$*$	
$M_\mathrm{X}$		$\checkmark$				$\checkmark$
$x_{I\!\!P}$	$\checkmark$	$\checkmark$	$\checkmark$	$\checkmark$	$\checkmark$	$\checkmark$
$z^\mathrm{obs}_{I\!\!P}$	$\checkmark$	$\checkmark$	$\checkmark$	$\checkmark$		$\checkmark$
$(Q^2;p_{\mathrm {T}}^{*,\mathrm jet1})$			$\checkmark$			
$(Q^2;z^\mathrm{obs}_{I\!\!P})$			$\checkmark$			$\checkmark$
$(p_{\mathrm {T}}^{*,\mathrm jet1};z^\mathrm{obs}_{I\!\!P})$						$\checkmark$

**Fig. 5:**
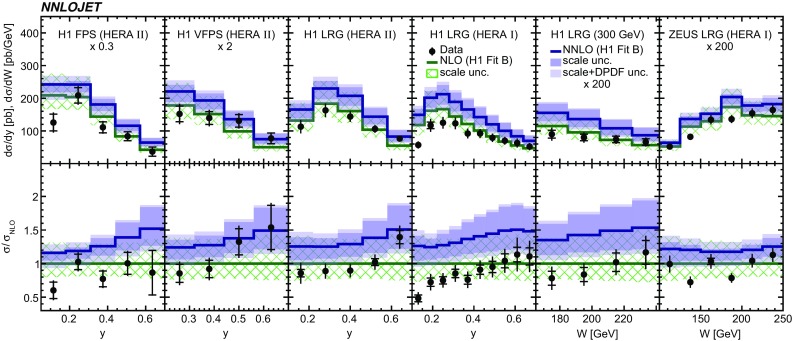
The differential cross sections as a function of *y* or, equivalently, *W*. In the upper panel, some of the distributions are scaled by a constant factor for better visibility. Displayed are the NNLO predictions in comparison to data and NLO predictions. The lower panel displays the ratio to NLO predictions. The shaded (hatched) area indicates the scale uncertainty of the NNLO (NLO) predictions. The bright shaded area around the NNLO predictions displays the scale and DPDF uncertainty added in quadrature

**Fig. 6:**
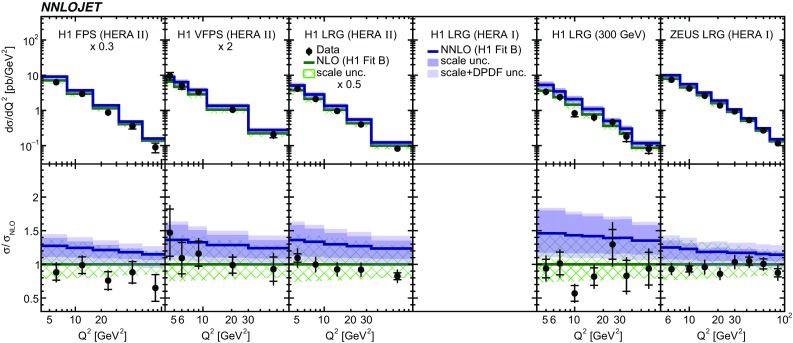
The differential cross sections as a function of $Q^2$. In case where the panel is empty, the respective analysis did not provide a measurement of the displayed observable. Other details as in Fig. 5

**Fig. 7:**
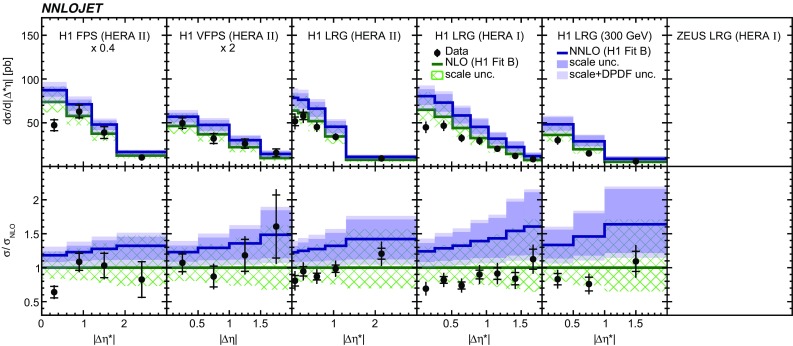
The differential cross sections as a function of $|\Delta \eta ^{*} |$ or $|\Delta \eta |$. Other details as in Fig. 5

**Fig. 8:**
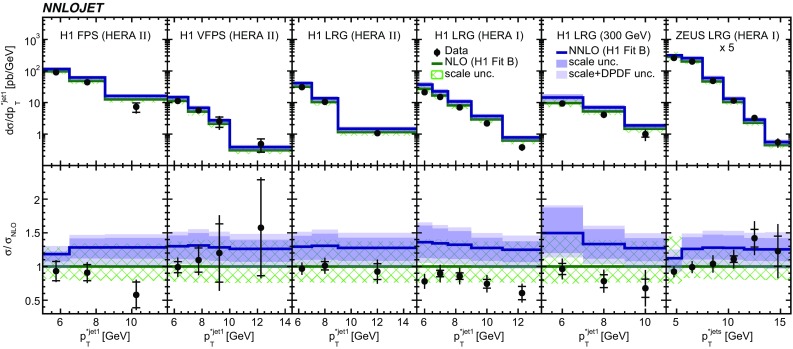
The differential cross sections as a function of $p_{\mathrm {T}}^{*,\mathrm jet1}$ or $p_{\mathrm {T}}^{*,\mathrm jet}$. Other details as in Fig. 5

**Fig. 9:**
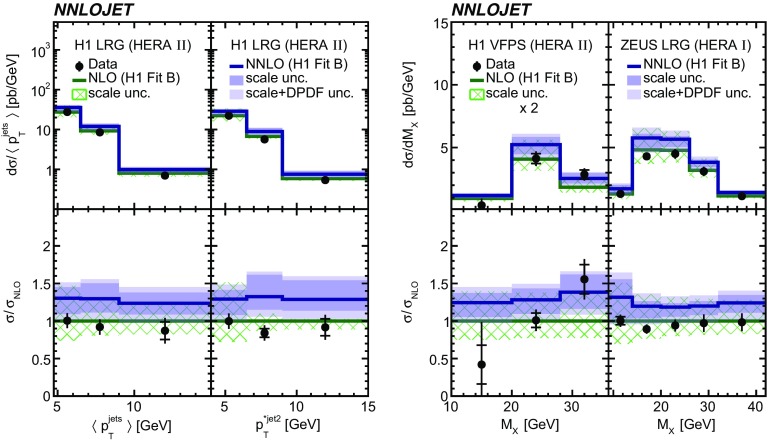
The differential cross sections as a function of $\langle p_{\mathrm {T}}\rangle$ and $p_{\mathrm {T}}^{*,\mathrm jet2}$ as measured in H1 LRG (HERA 2) (left), and as a function of $M_\mathrm{X}$ as measured in H1 VFPS (HERA 2) and ZEUS LRG (HERA 1) (right). Other details as in Fig. 5

**Fig. 10:**
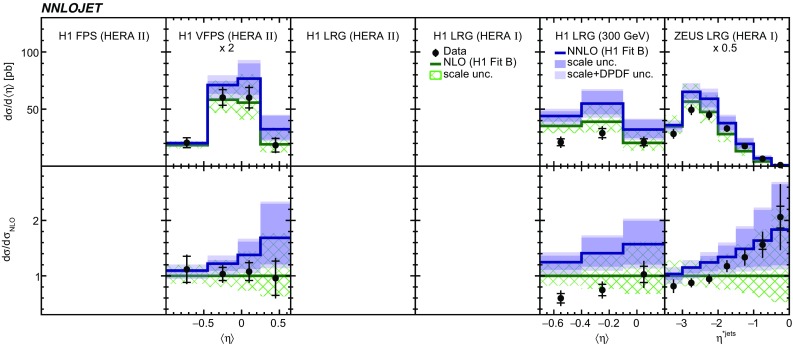
The differential cross sections as a function of $\langle \eta _\mathrm{lab}^\mathrm{jet}\rangle$ or $\eta _\mathrm{jet}^{*}$. Other details as in Fig. 5

**Fig. 11:**
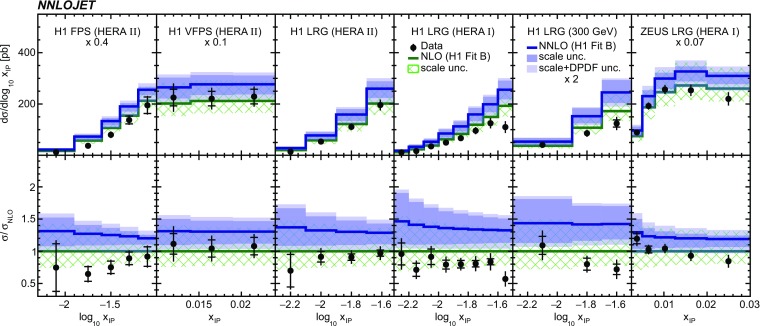
The differential cross sections as a function of $x_{I\!\!P}$. Other details as in Fig. 5

**Fig. 12:**
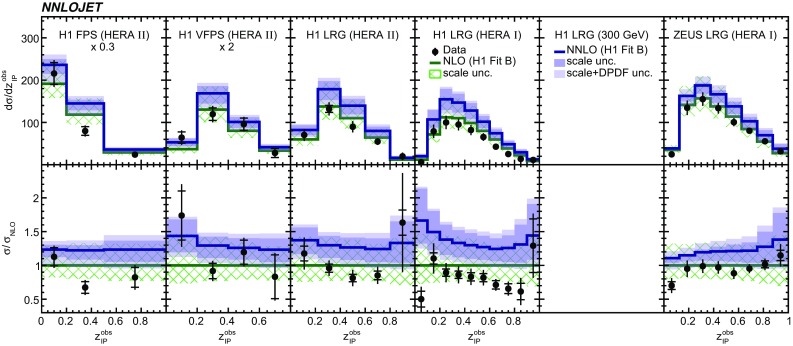
The differential cross sections as a function of $z^\mathrm{obs}_{I\!\!P}$. Other details as in Fig. 5

**Fig. 13:**
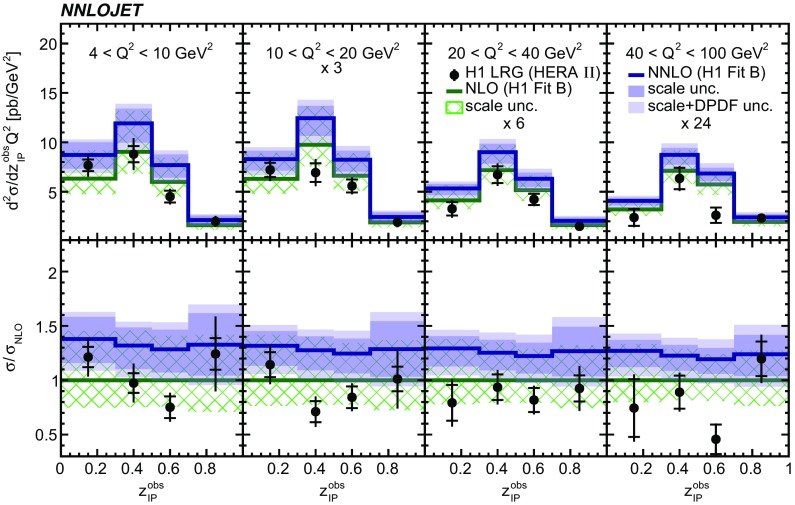
The double-differential cross sections as functions of $z^\mathrm{obs}_{I\!\!P}$ and $Q^2$ as measured in H1 LRG (HERA 2). Other details as in Fig. 5

**Fig. 14:**
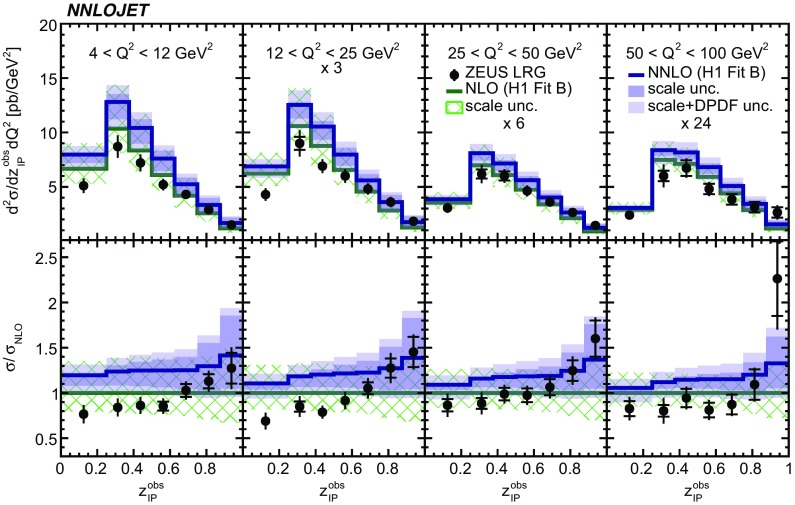
The double-differential cross sections as functions of $z^\mathrm{obs}_{I\!\!P}$ and $Q^2$ as measured in ZEUS LRG (HERA 1). Other details as in Fig. 5

**Fig. 15:**
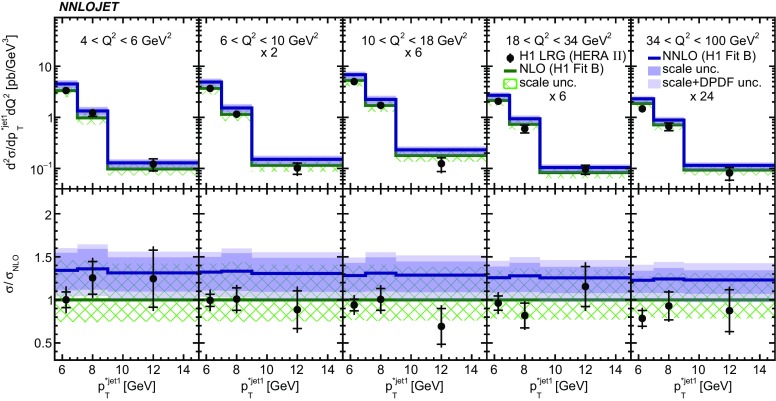
The double-differential cross sections as functions of $p_{\mathrm {T}}^{*,\mathrm jet1}$ and $Q^2$ as measured in H1 LRG (HERA 2). Other details as in Fig. 5

### Differential distributions

In total we computed 40 single-differential distributions and four double-differential distributions for available measurements, which are summarised in Table 5.

The NNLO predictions and their ratio to NLO predictions as a function of the inelasticity *y* are displayed together with their experimental data in Fig. 5. The inelasticity *y* is related to the $\gamma ^*p$ centre-of-mass energy by $W\simeq \sqrt{ys}$. The NNLO predictions provide an improved description of the shape of the data compared to respective NLO predictions, while being too high in their normalisation. The NNLO scale uncertainty is significantly reduced in comparison to the NLO scale uncertainty, which is most distinct at lower values of *y*.

**Fig. 16:**
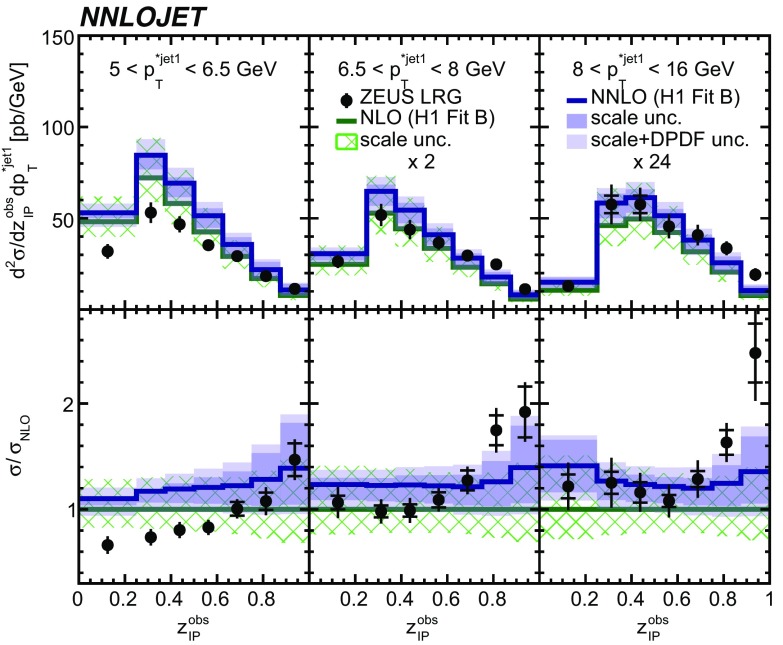
The double-differential cross sections as functions of $z^\mathrm{obs}_{I\!\!P}$ and $p_{\mathrm {T}}^{*,\mathrm jet1}$ as measured in ZEUS LRG (HERA 1). Other details as in Fig. 5

**Fig. 17:**
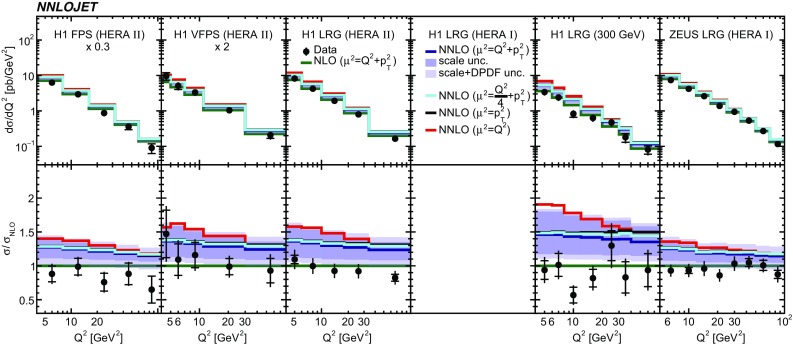
The differential cross sections as a function of $Q^2$. Displayed are NNLO predictions for different scale definitions. Further details are given in Fig. 5

**Fig. 18:**
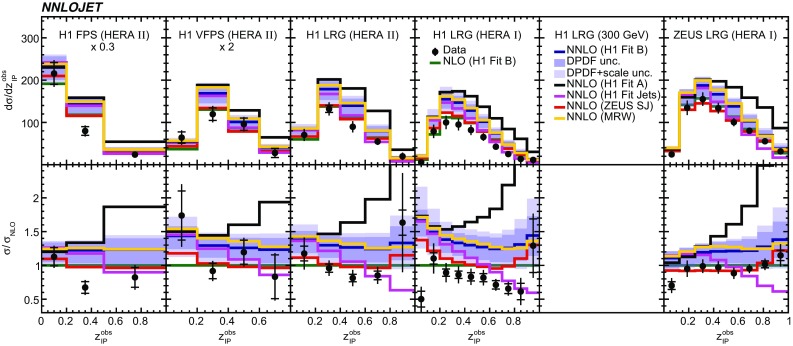
The differential cross sections as a function of $z^\mathrm{obs}_{I\!\!P}$. Displayed are NNLO predictions for different DPDFs. Further details are given in Fig. 5

**Fig. 19:**
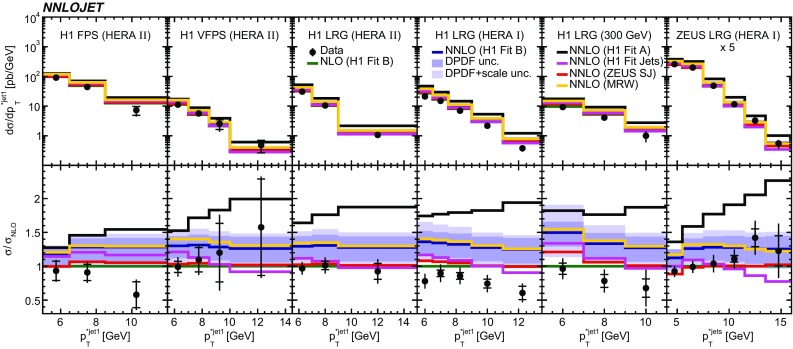
The differential cross sections as a function of $p_{\mathrm {T}}^{*,\mathrm jet1}$ or $p_{\mathrm {T}}^{*,\mathrm jet}$ obtained for different DPDFs. Other details as in Fig. 5

**Fig. 20:**
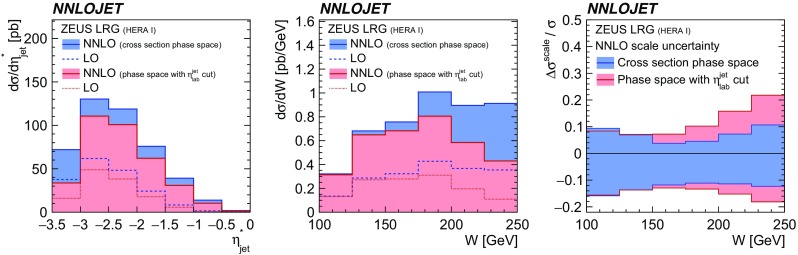
NNLO and LO predictions for the ZEUS LRG (HERA 1) phase space with and without the additional cut of $-1<\eta _\mathrm{lab}^\mathrm{jet} <2.5$ for two selected observables: $\eta _\mathrm{jet}^{*}$ (left) and *W* (middle). The right panel displays the relative NNLO scale uncertainty for the *W* distribution for the two studied phase space definitions

The NNLO predictions as a function of $Q^2$, $|\Delta \eta ^{*} |$ (or $|\Delta \eta |$), $p_{\mathrm {T}}^{*,\mathrm jet1}$ (or $p_{\mathrm {T}}^{*,\mathrm jet}$), $\langle p_{\mathrm {T}}\rangle$, $p_{\mathrm {T}}^{*,\mathrm jet2}$, $M_\mathrm{X}$, $\langle \eta _\mathrm{lab}^\mathrm{jet}\rangle$ (or $\eta _\mathrm{jet}^{*}$), $x_{I\!\!P}$^4^ and $z^\mathrm{obs}_{I\!\!P}$ are presented in Figs. 6, 7, 8, 9, 10, 11 and 12, respectively, and compared to data. Double-differential predictions as functions of $z^\mathrm{obs}_{I\!\!P}$ and $p_{\mathrm {T}}^{*,\mathrm jet1}$ for $Q^2$ intervals, and as a function of $z^\mathrm{obs}_{I\!\!P}$ for $p_{\mathrm {T}}^{*,\mathrm jet1}$ intervals are presented in Figs. 13, 14, 15 and  16. Similar conclusions as for the *y* distribution can be drawn from these comparisons. Some variants of selected distributions are discussed in more detail in the following.

While *y* is an inclusive observable, the rapidity separation of the two leading jets, $|\Delta \eta ^{*} |$, is directly sensitive to effects emerging from higher order radiative corrections. Also for this observable, the NNLO predictions provide an improved description of the shape for measured distributions, as can be seen in Fig. 7. Similar observations are made for all remaining distributions. This in particular for distributions in $Q^2$, $\langle \eta \rangle$ and $z^\mathrm{obs}_{I\!\!P}$  (see Figs. 6, 10,  12).

NNLO predictions as a function of $Q^2$ obtained with different scale definitions are displayed in Fig. 17. For this study we set $\mu := \mu _F = \mu _R$. The studied scale definitions $\mu ^2 = Q^2/4 + \langle p_{\mathrm {T}}\rangle ^2$ and $\mu ^2 = \langle p_{\mathrm {T}}\rangle ^2$ provide similar results as the nominal scale definition of $\mu ^2 = Q^2 + \langle p_{\mathrm {T}}\rangle ^2$, whereas the scale choice $\mu ^2 = Q^2$ results in higher cross sections and a steeper $Q^2$ spectrum. The studied scale choices are covered by the scale uncertainties.

NNLO predictions for $z^\mathrm{obs}_{I\!\!P}$ distributions obtained using different DPDFs are displayed in Fig. 18. For this observable, NNLO predictions using the H1FitB and MRW DPDFs give quite similar results and lie above most of the data. Results obtained with the H1FitA DPDF significantly overestimate the measurements in particular for higher values of $z^\mathrm{obs}_{I\!\!P}$. Predictions obtained with ZEUSSJ and H1FitJets give lower cross sections, but the application of the H1FitJets DPDF also results in a considerably different shape of the distribution. In general, the latter two DPDFs, including dijet data in their determination, give an improved description of the data compared to the first two DPDFs. It should be noted however, that differences arising from applications of different DPDFs are not covered by the uncertainties taken from the H1FitB DPDF. This feature is most prominent at higher values of $z^\mathrm{obs}_{I\!\!P}$.

In summary, NNLO predictions using the stated DPDFs provide an overall satisfactorily description of the data. However, none of the studied DPDFs is able to describe the shapes of the distributions of all of the $p_{\mathrm {T}}^{*,\mathrm jet1}$ (or $p_{\mathrm {T}}^{*,\mathrm jet}$) measurements equally well, as can be seen from their comparisons to predictions displayed in Fig. 19.

The studied DPDFs mainly differ in their gluon component [65]. This explains the observed differences between results obtained with different DPDFs as the gluon is the most important parton inside the DPDFs. It is therefore crucial to determine the gluonic component of the DPDFs more accurately, and once this is achieved, theoretical predictions are expected to provide an improved description of the data.

Despite the fact that the H1 and ZEUS experimental devices have a similar resolution and comparable acceptances, it is observed that predictions for the ZEUS LRG (HERA 1) phase space often yield smaller scale uncertainties as those for the comparable H1 LRG (HERA 2) phase space. This is mainly due to the restriction on $\eta _\mathrm{lab}^\mathrm{jet}$ imposed by H1, whereas the ZEUS phase space is restricted only in $\eta _\mathrm{jet}^{*}$, even though an equivalent requirement on $\eta _\mathrm{lab}^\mathrm{jet}$ is imposed for ZEUS LRG (HERA 1) measurement on detector level [62]. In Fig. 20 a study is presented, where an additional $\eta _\mathrm{lab}^\mathrm{jet}$ cut of $-1<\eta _\mathrm{lab}^\mathrm{jet} <2.5$ on the NNLO and LO predictions for the ZEUS LRG (HERA 1) phase space is shown.^5^ In particular at lower values of $|\eta _\mathrm{jet}^{*} |$ and at higher values of *W*, this cut would significantly reduce the cross section.

**Fig. 21:**
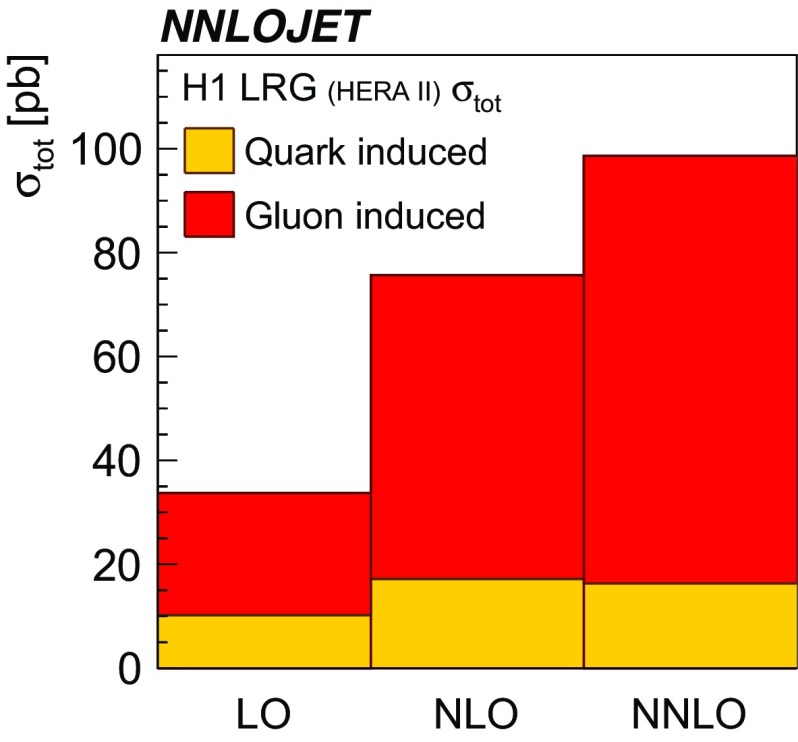
The decomposition of the H1 LRG (HERA 2) total dijet cross section into the part induced by gluons (red) and quarks (yellow). It is shown at LO, NLO and NNLO

**Fig. 22:**
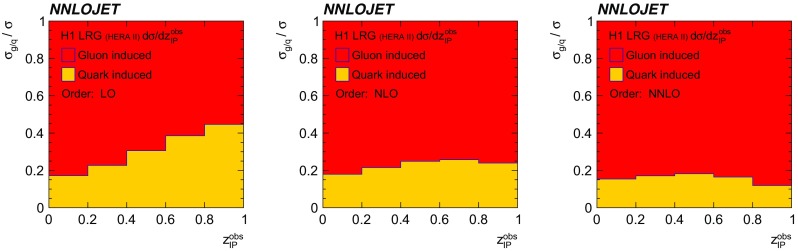
The fraction of gluon induced (red) and quark induced (yellow) contributions of the LO (left), NLO (middle) and NNLO (right) cross section as a function of $z^\mathrm{obs}_{I\!\!P}$. The kinematic range is adapted from the H1 LRG (HERA 2) measurement

Once the additional cut on $\eta _\mathrm{lab}^\mathrm{jet}$ is imposed, the relative NNLO scale uncertainty increases significantly, i.e. up to a factor of two in some parts of the phase space. This becomes in particular distinct at high values of *W*, as displayed in Fig. 20 (right). In conclusion, it is observed that the phase space definition of ZEUS LRG (HERA 1) results in more stable pQCD predictions, i.e. lower scale uncertainties, while important regions of the phase space were not accessible by the experimental device and the extrapolation factors were obtained by MC simulations. Similar considerations also apply to the H1 LRG (HERA 1) measurement.

### The gluon induced fraction

In order to further elucidate the dependence of the NNLO predictions on the individual parton flavors inside the DPDFs, the decomposition of the total H1 LRG (HERA 2) cross section into gluon-induced and quark-induced channels is shown for LO , NLO and NNLO predictions in Fig. 21. It is apparent that the rise of the cross section at higher orders is predominantly driven by the gluon-induced channels.

The fractions of gluon- and quark-induced contributions to the cross sections as a function of $z^\mathrm{obs}_{I\!\!P}$ are displayed in Fig. 22. While the fraction of the gluon-induced contribution remains unchanged for different orders in $\alpha _s$ at low values of $z^\mathrm{obs}_{I\!\!P}$, there is a strong increase of the gluon-induced fraction at higher values of $z^\mathrm{obs}_{I\!\!P}$ for higher orders in $\alpha _s$. Hence it can be deduced that future NNLO DPDFs are required to have a significantly reduced gluon component as compared to currently available NLO DPDFs.

### The sensitivity to DPDFs

**Fig. 23:**
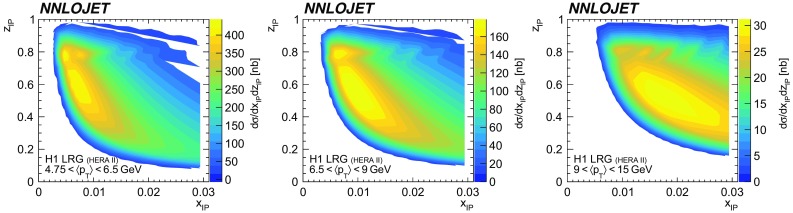
Contributions to the cross section for $\mathrm {d}\sigma /\mathrm {d}\langle p_{\mathrm {T}}\rangle$ of the H1 LRG (HERA 2) measurement as a function of $x_{I\!\!P}$ and $z_{I\!\!P}$ (bin integrated). The three pads represent the three bins of this measurement. The color coding represents the differential cross sections as function of $z_{I\!\!P}$ and $x_{I\!\!P}$ on a linear scale. The white areas are kinematically forbidden

A detailed study on the dependence of the cross section on the DPDF is presented for the $\langle p_{\mathrm {T}}\rangle$ distribution of the H1 LRG (HERA 2) measurement. The contributions to the cross section in each bin as a function of the DPDF parameters $x_{I\!\!P}$ and $z_{I\!\!P}$ is displayed in Fig. 23. At highest values of $\langle p_{\mathrm {T}}\rangle$, only partons with comparably high values of $x_{I\!\!P}$ and $z_{I\!\!P}$ are contributing to the cross section, whereas the cross section at medium values of $\langle p_{\mathrm {T}}\rangle$ is dominated by low $x_{I\!\!P}$ and $z_{I\!\!P}$ partons. All three bins have recognisable contributions from high values of $z^\mathrm{obs}_{I\!\!P}$ which is a distinct feature for predictions obtained with the H1FitB DPDF.

### Quantitative comparison

**Fig. 24:**
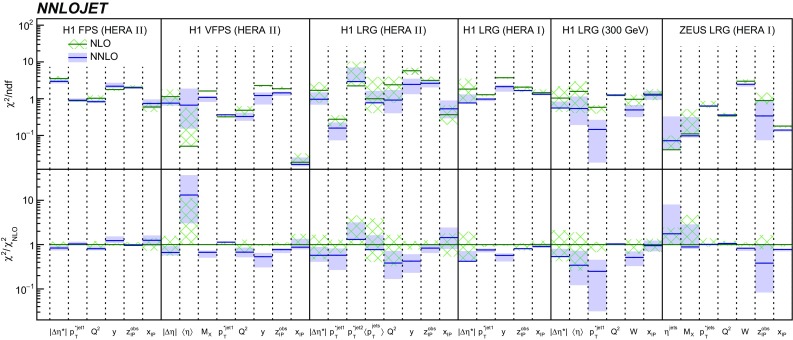
The $\chi ^{2}/n_\mathrm{dof}$ values for the analysed single-differential distributions obtained with NNLO and NLO predictions. The lower panel displays the ratio of $\chi ^{2}/n_\mathrm{dof}$ to the NLO result. The size of the bands correspond to scale uncertainties. In all cases the H1FitB DPDF was used

The agreement of NLO and NNLO predictions with data is quantified in terms of a $\chi ^{2}$ test. The $\chi ^{2}$ function is defined as [66]

10 $$\begin{aligned} \chi ^{2} = \sum _{i,j} \log \tfrac{\sigma _i^\mathrm{Data}}{\sigma _i^\mathrm{(N)NLO}} (V^{-1})_{ij} \log \tfrac{\sigma _j^\mathrm{Data}}{\sigma _j^\mathrm{(N)NLO}}\,, \end{aligned}$$

where the predictions $\sigma _{i,j}^\mathrm{(N)NLO}$ and data $\sigma _{i,j}^\mathrm{data}$ for all points (*i* or *j*) of a differential distribution are considered and *V* denotes the covariance matrix calculated from the relative experimental uncertainties. We consider systematic uncertainties as fully correlated, if not stated differently in the original publication. In order to quantify only the agreement in shape, we consider the normalisation as a free parameter and minimise $\chi ^{2}$ with respect to it. We calculate $\chi ^{2}$ for all analysed single-differential distributions. Results for $\chi ^{2}/n_\mathrm{dof}$ are displayed in Fig. 24. For most of the distributions the $\chi ^{2}/n_\mathrm{dof}$ values are smaller when using NNLO rather than NLO predictions.

The calculations are repeated for different DPDFs and different scale functional forms and also in these cases, it is observed that NNLO predictions mostly give lower $\chi ^{2}/n_\mathrm{dof}$ values than NLO predictions (not shown). In an approximation, the normalisation of the predictions is proportional to the gluon content of the DPDFs, whereas the shapes of the differential distributions are related more closely to the hard matrix elements. Therefore, these results indicate that NNLO predictions provide a better description of the data than NLO predictions, and we believe that future DPDFs determined to NNLO QCD will be able to provide an improved description of the dijet data, this also with respect to the normalisation.

From the double-differential distributions, we select the $\mathrm {d}\sigma /\mathrm {d}Q^2 \mathrm {d}p_{\mathrm {T}}^{*,\mathrm jet1}$ measurement of the H1 LRG (HERA 2) analysis, and data are compared to the NNLO and NLO predictions in Fig. 15. For the $\chi ^{2}$ evaluation, we minimise $\chi ^{2}$ as a function of $\alpha _{\text {s}} (M_{\text {Z}})$, which is an equivalent procedure to the $\alpha _{\text {s}} (M_{\text {Z}})$ determination presented previously by H1 [3]. The calculation using NLO predictions results in $\chi ^{2}/n_\mathrm{dof} =16/14$. The calculation using NNLO predictions results in a value of $\chi ^{2}/n_\mathrm{dof} =13/14$, thus indicating also in this case an improved description of the data. We estimate a scale uncertainty on the best fit value of $\alpha _{\text {s}} (M_{\text {Z}})$ with additional calculations using scale factors of 0.5 and 2 6. The scale uncertainty of $\alpha _{\text {s}} (M_{\text {Z}})$ is found to be 11 % for the NLO predictions, and for the NNLO predictions it is reduced to 4 %. This reduction quantifies the significant improvement of the NNLO predictions as compared to NLO predictions. The NNLO scale uncertainty is of similar size as the experimental one or the DPDF uncertainties on $\alpha _{\text {s}} (M_{\text {Z}})$, where H1 reported 4 % for both [3]. This study demonstrates that the NNLO calculations are suitable for further phenomenological analyses, such as $\alpha _{\text {s}} (M_{\text {Z}})$ or DPDF fits, and the NNLO scale uncertainties are of equal size as experimental uncertainties.

## Discussion and summary

We present the first NNLO QCD predictions for jet production in diffractive scattering. Predictions for six measurements of dijet production in diffractive deep-inelastic scattering from the H1 and ZEUS collaborations were calculated and compared to data. We observe that the NNLO cross sections are significantly higher than the data and are higher than NLO calculations by about 20–40 % in the studied kinematical range.

Since no DPDFs in NNLO accuracy are available so far, only NLO DPDFs could be employed for our calculations. The discrepancy of the NNLO predictions and data is believed to be due to an overestimated gluon component of these DPDFs. Alternative DPDFs, which also considered dijet data in their determination, already result in typically lower NNLO predictions, but these still overshoot the data. Ignoring the issue of normalisation, the shapes of differential distributions are better described by NNLO than NLO predictions. This is quantified by evaluating $\chi ^{2}$ values for the examined experimental distributions. The large amount of studied observables, which have so far not even been studied in non-diffractive DIS, prove that NNLO predictions provide an improved description in shape of the data throughout.

We believe that the normalisation difference between data and NNLO predictions can be resolved by employing DPDFs determined to NNLO accuracy and by including dijet data for their determinations. This in particular as the gluon component is most important and is only weakly constrained by the inclusive data. The NNLO dijet calculations presented here are already in a numerical format, which is suitable for such future analyses.

The comprehensive selection of all available dijet data represents the first comparison, where all these measurements are compared to predictions obtained in an identical framework. Data taken with different experimental devices, at different center-of-mass energies, and using either proton spectrometers or the LRG method for the identification of the diffractive final state are investigated. All measurements are found to be mutually consistent when compared to respective predictions.

The presented NNLO predictions provide the most precise predictions for dijet production in diffractive DIS to date, and the dominant theoretical uncertainty is reduced substantially with respect to predictions in NLO. The NNLO coefficients exhibit a precision in terms of scale uncertainties, which is of a comparable size as that of presently available DPDFs, and of comparable size as that of the HERA dijet data. It is observed, that for the given kinematical range of the HERA data, higher-order corrections are of crucial importance.

## Footnotes

1 Here, observables in the $\gamma ^*p$ (laboratory) frame are conventionally denoted with an asterisk ‘$^*$’ (superscript ‘lab’).

2 In inclusive DDIS the invariant $\beta ={Q^2}/{2q\cdot (p-p')}$ has a similar interpretation, which can also be calculated as $\beta = {x_{\mathrm {Bj}}}/{x_{I\!\!P}}$, with $x_{\mathrm {Bj}} ={Q^2}/{2 p\cdot q}$.

3 Although, the commonly employed approach for the determination of the DPDFs is a global fit to experimental data, the DPDFs can alternatively be defined through light-cone matrix elements, and in this framework certain aspects of their behaviour at the starting scale of the evolution can be computed [47].

4 The numerical values of the $x_{I\!\!P}$ distribution of the ZEUS LRG (HERA 1) measurement were provided to us by the ZEUS physics office [64].

5 The ZEUS LRG (HERA 1) analysis required two jets to be within $-2<\eta _\mathrm{lab}^\mathrm{jet} <2$ [62]. For better comparability and also due to technical reasons, we study an additional cut of $-1<\eta _\mathrm{lab}^\mathrm{jet} <2.5$ in analogy to the H1 FPS (HERA 2) and H1 VFPS (HERA 2) measurements.

6 The H1 collaboration estimated an uncertainty for $\mu _{\text {R}}$ and $\mu _{\text {F}}$ separately and considered the resulting uncertainties on the cross sections as half correlated and half uncorrelated.
